# A Multi-Perspective Framework of Vision Zero: Toward Collaborative Promotion of Safety, Health and Well-Being at Work

**DOI:** 10.1016/j.shaw.2022.05.001

**Published:** 2022-05-07

**Authors:** Tommi Alanko, Riikka Ruotsala

**Affiliations:** Finnish Institute of Occupational Health, Finland

**Keywords:** Vision Zero, collaboration, multi-perspective framework, safety health and well-being at work

## Abstract

In the globalized field of safety, health, and well-being, the need to build multi-stakeholder alliances to find solutions to complex challenges is growing. This requires common ground for collaboration, as well as concepts and tools to grasp and manage the areas of interest. Over recent years, Vision Zero has awakened interest and it continues to evolve into many forms of approaches and initiatives, which provide a strategic direction and practical tools for supporting the development of safety, health, and well-being at work. Consequently, there is a need to build a shared understanding of ‘zero’. This article reflects the potential of Vision Zero as a bridging concept and an approach for building multi-stakeholder collaboration. Thus, we present a multi-perspective framework of Vision Zero to support further dialoge and collaboration in joint undertakings.

## 1 Introduction

Zero-related terminology has a decades-long history in safety-related fields. The very concept of Vision Zero (VZ) originates from the Swedish road traffic safety, from which it has expanded to international road traffic safety policy and to other accident prevention areas [1,2]. In recent years, VZ has awakened increasing interest in occupational health and safety (OSH) and has gained ground as a prevention strategy. The focus is mainly not only on how to establish and develop a prevention culture in enterprises but also more widely in networks across sectors, as well as at national [3], and global levels [4,5].

Here, we comprehend Vision Zero as a concept and approach that features the aspects of safety, health, and well-being (SHW) at work. In work organizations, this relates to the view that ‘VZ should be regarded as a holistic vision, wherein health, safety, and well-being at work are all addressed, and synergies between these areas are recognized and utilized*’* [6]. Well-being at work, although there is no consensus on its definition, expands the focus from the absence of negative consequences, such as accidents and diseases, to the mental and social dimensions of work [7]. As it also includes positive features related to work and personal life, well-being at work is seen as a determinant of productivity at the individual, enterprise, and societal level [8]. Along these lines, we relate VZ to the continuous development and improvement of SHW at work, which encompasses both effective measures to *prevent* work-related accidents and diseases and innovative practices to *promote* SWH at different levels of policy and practice. Further, we view that, VZ values human life and underlines ethical business and work life principles: the implementation of VZ should not have consequences that weaken anyone's SHW at work, neither in workplaces nor in wider supply chains.

As Vision Zero continues to evolve in many forms of global and national initiatives and strategies, as well as sector- and company-level programmes, the need to build a shared understanding of the concept is growing: What does VZ stand for and how it is applied among the wide variety of stakeholders such as business and sustainability managers, OSH practitioners, policymakers, NGOs, authorities, and academics? By shared understanding, we do not mean that there is, or should be, one universal definition of or approach to VZ. On the contrary, we maintain that the potential of VZ lays in the synergy that aligning different perspectives generates, in terms of both the prevention of accidents and the promotion of SHW at work.

In this article, we view Vision Zero in the context of building multi-stakeholder collaboration in the changing world of work. First, we explore the zero terminology and different conceptualizations. Second, we contemplate whether VZ—as a concept and joint approach—could provide a common ground for aligning SHW at work with the wider agenda of sustainable development. As a result, we outline a multi-perspective framework of VZ to support dialoge and collaboration in joint undertakings.

## 2 Vision Zero conceptualizations

Vision Zero is an example of the diverse set of zero conceptualizations used to express and outline various development scopes and prevention objectives across several fields. In general, practitioners and academics have debated the pros and cons of zero-related terminology. We acknowledge the existing critics, particularly of zero in the safety field [6,9,10], by roughly touching upon them, but they are not the focus of this article. We believe that more attention should be paid to the conceptual terrain of VZ because there is a risk that its potential may be ‘lost in translation’.

As an example, the term Vision Zero is conceptualized in different contexts as a principle, a vision, a mindset, an approach, a policy, a strategy, a goal, a campaign, and a programme—respectively, accompanied by more descriptive words such as long-term, transformative, ethical, holistic, and strategical. Consequently, in a conversation for instance, one might approach VZ from the *strategical* perspective and comprehend it as an overarching vision to *promote* SHW, whereas another might refer to statistics to illustrate the quantified goals in accident *prevention,* and a third person might recognize it as *practical* tools and methods.

The rough example points out the diverse set of meanings that might be attached to one concept. This relates to the characterization that concepts are dynamic, and partially conflicting conceptualizations may emerge as a result of the collective concept formation processes [11]. Inevitably, the wide-ranging purposes in which the concept is used encompass different agendas and background motives with temporally varying emphases. This should be considered in collaborative settings when taking a stance toward ‘zero’. As the conceptualizations may bear multiple meanings, an imprecise use of words may lead to unintended misinterpretations, and as a result, discussions might become counter-productive: the concept remains vague and is deployed as partly optimized.

As a starting point for forming joint approaches, stakeholders should explicate their rationales and guiding principles. Complex concepts have been described as products and tools of collective activities that evolve historically, and the concepts convey ethical and ideological aspects and mediate emotions [11]. Respectively, various ‘zero’ initiatives and approaches have different historically evolved paths, it is hard to comprehend what they share or in which terms they differ. In recent years, the conceptual umbrella of ‘zero’ has expanded, and currently, many conceptualizations are linked to sustainability aspirations. For example, zero emissions, zero waste, and zero pollution highlight environmental aspects. In business, ‘zero’ has been increasingly branded as underlining sustainable corporate strategies and product development. In OSH vocabulary, ‘zero harm’ and ‘zero accidents’ have their own development paths and areas of application [6].

Zero terminology has also been a polemic in the academic arena. In relation to workplace safety, it has been critically debated whether ‘zero’ frames a rational or irrational goal. This line of critics questions the constraints of comprehending the concept as a quantified target of ‘zero vision’, ‘zero accident’, which for instance includes a risk of hiding and manipulating incident data, putting emphasis on counting negatives and missing opportunities for learning [9,10]. This points out a discrepancy between the ideal, unachievable target, and the reality, existing daily practices [12]. In counterarguments, it has been reasoned that ‘zero’ should be approached as a process and the outcomes should be evaluated in relation to its implementation, not numbers per se [6]. Zwetsloot [13] distinguishes between ‘theory failure’ and ‘implementation failure’ by pointing out that striving for zero requires ‘doing things right’. He further states that “if ‘zero’ is espoused merely as a rhetoric slogan, it is, of course, not credible for the workers who experience in every day practice that it changes nothing or very little” [13, p. 120]. Both lines of reasoning seem to underline the same weak spot; the gap between strategical principles and practical action. Similarly, they view the importance of participation of people and renewal of safety management and practices. This indicates that ‘zero’ needs to be embedded in activity and approached and evaluated from several perspectives.

Concepts and reasoning are the core of academic discussion and new knowledge is constructed by questioning and rationalizing different points of view, hypotheses, and theories. In this sense, these debates are very much an ongoing concept formation apparatus, and in relation to zero conceptualizations, have introduced multiple perspectives and rationales. Overall, the concepts are socially constructed and new meanings are negotiated collectively [14].

## 3 Building multi-stakeholder collaboration in the changing world of work

In practice, concepts are learned when they are contested, re-constructed, and implemented [11]. Currently, the development of SHW at work is intertwined with globalization and the changing world of work. Work is increasingly performed in dynamic and systemic multi-organizational settings, such as global supply chains, which add complexity to managing OSH and promote decent work [15]. The contemporary challenges span from traditional OSH risks to questions of social responsibility and human rights [7]. Correspondingly, solutions are equally needed at the levels of workplace, industrial sector, society, and global networks. We comprehend that Vision Zero places prevention and promotion rationales in the dynamic field of SHW and relates it to a wider sustainable development agenda. Given this, SHW at work should not only be viewed as merely addressing problems and concerns but as also discovering opportunities for collective learning and development, a basis on which to build multi-stakeholder collaboration.

In general, the aspiration is to shift SHW at work onto a more comprehensive, transformative and sustainable development path. The United Nation's 2030 agenda and its sustainable development goals (SDGs) provides a map with which to navigate toward a better future [16]. SHW at work are interlinked to several SDGs and targets. They have strong links to, for instance, SDG 8 ‘Decent work and economic growth’ and SDG 3 ‘Healthy lives and well-being for all’. Stretching to resolve SHW concerns at all levels—to have a real impact—requires strengthening the existing forms of cooperation and creating novel alliances (cf. SDG 17 ‘Partnership for the goals’ [16,17]). In the literature, this is often addressed as a need for multi-disciplinary approaches and mainstreaming OSH into business management, which, in turn, entails crossing prevailing professional and functional boundaries. Linking SHW at work to a wider agenda of sustainable development should expand the sphere of involved stakeholders from OSH practitioners to other professional fields. For instance, aligning SHW with economics, public health and business ethics has been underlined [7].

As regards Vision Zero, we approach multi-stakeholder collaboration as a collective activity that crosses traditional boundaries and aims to build novel alliances and respond to new challenges. The question is how to enact this in practice?

We propose that Vision Zero could function as a conceptual bridge to catalyze collaboration. In fact, it has already brought people and organizations together globally and, hopefully, will continue to provide a platform for collaboration and collective learning. The following example presents how global initiatives have acknowledged the need for synergy.

*International Social Security Association* (ISSA) has launched a Vision Zero campaign. ISSA's approach is conceptualized as ‘Seven Golden Rules’, which form the basis, for instance, for guidebooks, training contents, and proactive leading indicators. ISSA's initiative is based on the enterprises' engagement, and by 2021, thousands of enterprises worldwide had joined the campaign [18].

The *Vision Zero* Fund is an initiative of the G7 and has been endorsed by the G20. The Vision Zero Fund is administered by the International Labour Organization and it aims to eliminate severe or fatal work-related accidents, injuries, and diseases in global supply chains [19]. Further, in *the* XXI World Congress on Safety and Health at Work in Singapore in 2017, a proposal for forming a *Global Coalition for Safety and Health at Work* (Global Coalition) was presented [20]. As a result, the Global Coalition was launched in 2019. One of the Global Coalition's task groups focuses on introducing Vision Zero to enterprises. In practice, the task group establishes a multi-stakeholder partnership, in which the two above-mentioned global initiatives, together with other stakeholders, work together to foster VZ collaboration [17].

## 4 Multi-perspective framework of Vision Zero

**Fig. 1:**
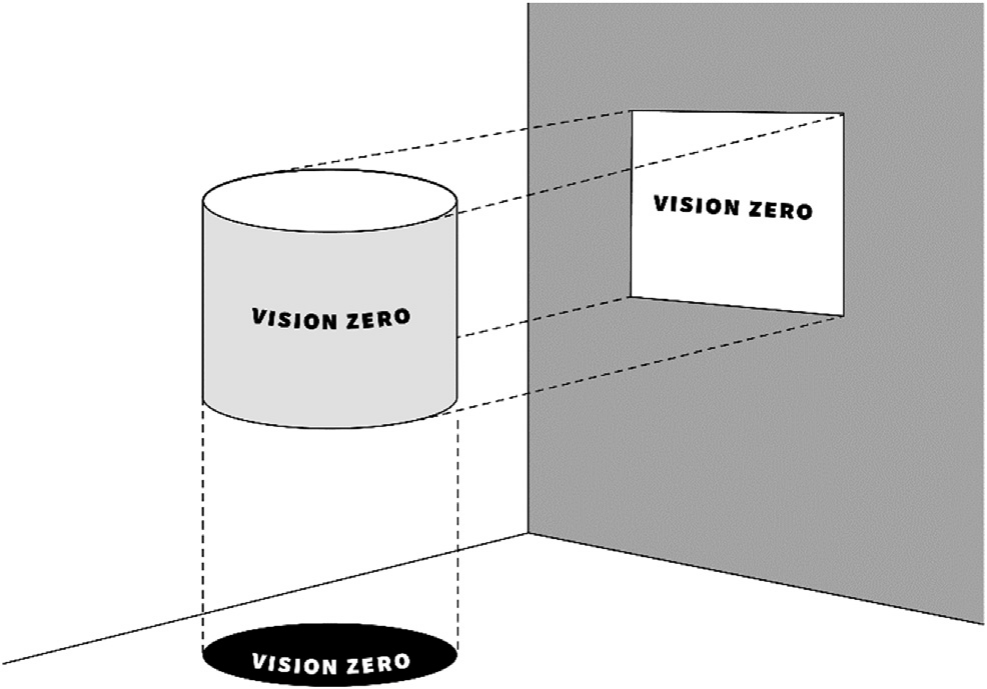
Multi-perspective framework of Vision Zero [21].

To overcome the potential conceptual pitfalls and implementation barriers and to support the creation of a common ground and shared understanding in joint undertakings, we present a multi-perspective framework for Vision Zero (Fig. 1). The framework is visualized as a cylinder that creates shadows which have different forms. The illustration presents a simplified view of how VZ, as a multi-dimensional entity (a cylinder), conveys different perspectives (shadows). The shadows exemplify that the way in which Vision Zero is perceived, comprehended, and conceptualized, depends on the perspective taken. Respectively, it can be argued that all manifestations or perspectives of VZ are equally correct and true.

The multi-perspective framework of Vision Zero provides a tool for building a shared understanding and common ground. The framework should be applied in relation to *collaborative* and *temporal* dimensions. First, it can be utilized in collaborative and multi-stakeholder settings. For instance, it could benefit stakeholders by aligning perspectives when forming networks, cross-sectoral partnerships and joint approaches. Similarly, in organizational settings, the framework can support cross-functional collaboration; realizing joint undertakings under a wider zero umbrella, for instance, between management, production, sustainability strategy development, environment, occupational safety and health, and human resources.

The purpose of the illustration is not to shape Vision Zero into one format; on the contrary, the idea is to underline the importance of diverse perceptions and the need for multi-voiced collaboration. The practical implication of the framework is to support dialoge, explicate the background rationales, create shared agendas, and facilitate the collective negotiation of new meanings. The first step is to elucidate the perspectives and conceptualizations and to jointly consider how they might complement one another, thus providing a more comprehensive standpoint for collaboration. Or, in contrast, to make conclusions how the various perspectives might be partially conflicting or contradictory, which may, in turn, create confusion and result in counter-productive outcomes.

The temporal dimension, in turn, is essential for following how the Vision Zero concept and its implementation transform over time. As in any dynamic environment, conceptualizations evolve and meanings shift, and it is likely that multiple layers of past and current activities co-exist. Moreover, VZ approaches evolve alongside organizational transformations. For instance, VZ can function as a starting point for introducing a prevention strategy for an enterprise. Later, the enterprise might pursue a more comprehensive vision of SHW promotion and align the development with other areas of management. The adapted mindsets and conceptualizations in the organization require reframing. The multi-perspective framework now has two or more projections as shadows, which need to be jointly negotiated (Fig. 1). We argue that potentially conflicting views are, in fact, one-perspective snapshots of VZ. To avoid confusion among stakeholders, it is important to follow-up how matters develop over time, and evaluate the need to reframe practices, remodel tools and renegotiate concepts accordingly. The framework could also benefit in identifying potential gaps between strategy and practice or other relevant perspectives (cf. critics and reasoning presented in section 2). Applying the framework in evaluative manner would enable multi-voiced, collective learning in this respect.

## 5 Conclusion

Pursuing socially sustainable work life and decent work requires regenerative concepts and joint approaches. Addressing and resolving systemic issues in relation to SHW at work will increasingly require multi-professional expertise. In this article, we have reflected on VZ in the context of building multi-stakeholder collaboration in the changing world of work. We conclude that VZ bears the potential to connect actors and align perspectives. However, the VZ concept itself is complex and conveys multiple meanings. To build a common ground for collaboration, the concepts need joint negotiations of meanings, clear explicating of the stakeholders' aims and background motives and, eventually, to turn words into collective, transformative activity. To support this endeavor and to look beyond conceptual disputes, we depicted a multi-perspective *framework* for Vision Zero.

In the future, Vision Zero will call for research to gain more empirical and conceptual ground. To support practical implication, we need to examine the way in which multi-stakeholder collaboration emerges and what kind of impact the joint approaches generate. Is Vision Zero's potential as a bridging concept and a joint approach realized in practice?

As noted earlier, concepts provoke opinions and mediate emotions. As when building sustainable future, anti-cynicism and optimism about a better tomorrow is also needed for Vision Zero to make a real impact.

## Conflict of interest

The authors have no conflicts of interest.
